# Microstructure and Corrosion Behaviors of Gas Tungsten Arc Welds for Borated Stainless Steel Using Various Filler Metals

**DOI:** 10.3390/ma18030550

**Published:** 2025-01-25

**Authors:** Minseok Seo, Hyunbin Nam, Yongju Yoon, Namhyun Kang, Cheolho Park

**Affiliations:** 1 Department of Welding & Joining Science Engineering, Chosun University, Gwangju 61452, Republic of Korea; minseok0724@chosun.ac.kr (M.S.); hbnam12@chosun.ac.kr (H.N.); marine0382@chosun.ac.kr (Y.Y.); 2 Department of Materials Science and Engineering, Pusan National University, Busan 46241, Republic of Korea; nhkang@pusan.ac.kr

**Keywords:** borated stainless steel, gas tungsten arc welding, boron component, microstructure, corrosion behavior

## Abstract

In this study, the microstructure and corrosion behavior of gas tungsten arc (GTA) welds of borated stainless steel (BSS) with a boron content of 1.62 wt.% were investigated using various filler metals. The filler metals used in this study were 308L, 309L, and 310 without the B component. A small amount of the B component was observed in the weld metal (WM) of all specimens, even though none of the filler wires contained boron. This result was caused by the dilution of the B component from the BM into the WM by the welding heat. The segregation of boron in the WM resulted in Cr-depleted areas, which negatively affected the corrosion resistance of the welded specimens. The corrosion resistance of 308L WM with the highest fraction of B components was the most deteriorated, whereas 309L WM with the lowest boron content exhibited the best corrosion resistance. Using a filler metal without the B component is expected to effectively improve the weldability and corrosion resistance of BSS; however, it can also reduce the neutron absorption capacity. Therefore, for BSS to be used as a spent nuclear fuel storage container material, the boron content of the filler metal must be carefully considered. This study provides a foundation for research aimed at improving the development and applicability of filler metals in borated stainless steel and makes it competitive for application in fourth-generation nuclear power systems.

## 1. Introduction

Globally, nuclear power generation continues to increase, and the amount of spent nuclear fuel generated and accumulated is also increasing [1]. In this context, alloys with B and Gd components added to stainless steel have recently been developed to increase their storage capacity [2,3,4]. However, research on the interaction between key elements of stainless steel and Gd, as well as its microstructural behavior, remains insufficient, making structural materials with added B the primary choice for spent nuclear fuel storage containers. Borated stainless steel (BSS), which is also known as stainless steel with added boron, can absorb more thermal neutrons because of the B isotope [5,6]. Consequently, the packing density of spent nuclear fuel can be increased, and the manufacturing cost can be reduced by realizing both roles simultaneously without using the neutron absorber and structural materials separately [7]. BSS is used as a structural material in storage racks and transport containers designed to store spent nuclear fuel generated at nuclear power plants [8]. However, for safe usage, BSS must possess not only excellent thermal neutron absorption capability, but also sufficient mechanical properties and corrosion resistance [9,10,11].

The ASTM A887-89 standard specifies eight types of stainless steel based on AISI 304 with 0.20–2.25 wt.% B added, ensuring neutron shielding performance [12]. However, despite its excellent shielding performance, in austenitic stainless steel, most of the boron precipitates as coarse and irregularly shaped borides owing to its low solubility. Therefore, most researchers have focused on intensively studying the influence of boron, which is well documented. Moon et al. investigated the effects of boron content (0.2–1.76 wt.%) on the high-temperature ductility and solidification cracking susceptibility of borated stainless steel welds, reporting that increased boron content enhances resistance to solidification cracking but compromises hot ductility due to boride formation [13]. Ha et al. examined the pitting corrosion resistance of AISI 304-based borated stainless steels with boron contents of 0.19, 0.78, and 1.76 wt.% and observed that boride formation led to Cr segregation, reducing pitting resistance [14]. Wang et al. systematically studied the effects of electron beam welding (EBW) and post-weld heat treatment (PWHT) on the microstructure, mechanical properties, and fracture behavior of BSS with varying boron contents, highlighting that PWHT spheroidized borides to enhance ductility, while higher boron content increased strength but decreased ductility [15]. Won et al. investigated the microstructure and mechanical properties of BSS with different boron contents, focusing on the effects of annealing time using a thermodynamic approach, and concluded that although higher boron content reduces ductility, annealing refines boride morphology, thereby improving ductility [8]. In previous studies, the test methods were different; however, it is known that as boron increases, the phase fraction of borides increases, resulting in problems that greatly degrade the mechanical properties and corrosion resistance. However, because BSS is used as a structural material in the nuclear industry, stable corrosion resistance is essential to ensure long-term stability and reliability. In addition, to develop steel materials with excellent corrosion resistance, weldability evaluation must be performed.

However, in the case of BSS, the development of steel is actively taking place, but there has been almost no evaluation of the corrosion resistance to welding. Therefore, this study investigated the corrosion resistance according to the microstructural behavior of welds using three types of filler wires. To this end, a BSS with a boron content of 1.50 to 1.74 wt.% was welded using filler wires with different Cr contents, and the effect of the microstructure and component behavior of the weld on the corrosion resistance was evaluated.

## 2. Materials and Methods

### 2.1. Materials and Welding Methods

**Figure 1:**
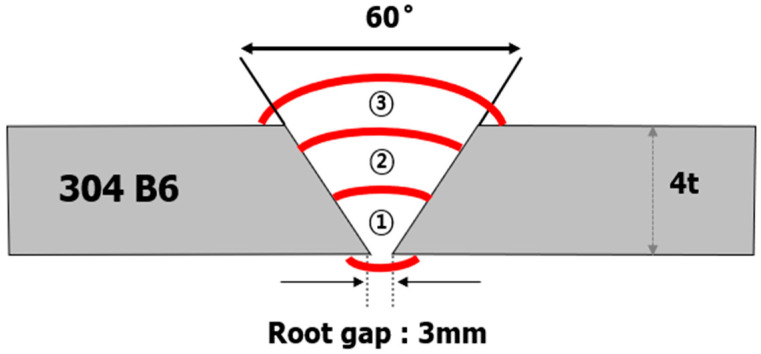
Schematic of the welded joint used in the present study.

In this study, three types of filler wires (ER308L, ER309L, and ER310) were employed to manufacture welding specimens with GTA welds applied to boron-containing stainless steel, which is an ASTM A887 Grade A Type 304B6 material. The compositions of the base metal (BM) and filler wires used in this study were analyzed by quantitative analysis of electron probe microanalysis (EPMA) and are shown in Table 1. It should be noted that the composition of each filler wire did not include boron, and the Cr content increased from ER308L to ER310. As for the welding conditions, as shown in Figure 1, only the first layer was subjected to a pulsed current to control the amount of heat input, and the same conditions were applied to the three types of filler wires. The detailed welding conditions are listed in Table 2.

### 2.2. Microstructure Analysis

To observe the microstructure of the weld, the specimen was polished to 1200 grit, and followed by wet polishing to 1 um using a diamond suspension. For microscopic observation, the specimens were etched using a mixed solution of 30 mL nitric acid, 20 mL hydrochloric acid, and 50 mL ethanol, and then observed using scanning electron microscopy (SEM: JSM-7900F, JEOL, Tokyo, Japan). Composition and segregation of the microstructure of the weld zone were observed using EPMA (JXA-8530F Plus, JEOL, Japan). Inductively coupled plasma (ICP) analysis was performed to confirm the boron content and overall composition of the diluted weld metal.

### 2.3. Corrosion Test

#### 2.3.1. Sensitization Test

Weld metal (WM) sensitization was measured using the ASTM A262 Practice A method [16]. This method is an electrochemical measurement method with the oxalic acid etch test, and before beginning the test, the specimen was polished to 1200 grit, and electrolytically etched under a 1 A/cm^2^ current for 90 s using an oxalic acid solution (100 g H_2_C_2_O_4_·2H_2_O + 900 mL distilled water) at room temperature. After the etch test, the surface structure of the specimen was observed under a microscope, and the tissue conditions were classified.

#### 2.3.2. Pitting Corrosion Test

Sensitivity to pitting corrosion was measured according to ASTM G48 Method E [17], and the estimated critical pitting temperature (CPT) was calculated. The experiment was conducted at an average temperature of 20 degrees. For this, the specimen polished to 1200 grit was immersed in a solution consisting of 68.72 g of ferric chloride (FeCl_3_·6H_2_O), 600 mL of distilled water, and 16 mL of hydrochloric acid for 24 h. After immersion, damage to the edges was ignored, and the shapes of the pits were observed under a microscope.

#### 2.3.3. Cyclic Polarization Test

For a quantitative evaluation, a cyclic potentiodynamic polarization test was conducted using a potentiostat (VersaSTAT300 DC corrosion technique, AMETEK Scientific Instruments, Oak Ridge, TN, USA). The tests were conducted in a three-electrode electrochemical cell with a Pt counter electrode, Ag/AgCl-3.5M KCl reference electrode, and the weld metal specimen as the working electrode. The working electrode specimen was polished to 2400 grit and then adjusted using a Teflon ring to expose only the weld area to 0.2826 cm^2^. Considering the low corrosion resistance of BSS, the test was conducted in a NaCl 50 ppm solution at 27 °C. Before beginning the experiment, cathodic cleaning was performed at −1 V for 500 s to remove the existing oxide film from the working electrode. Subsequently, the sample was immersed for 500 s for stabilization. The polarization potential began at −0.35 V in the forward direction, reached 1 V, and then proceeded in the reverse direction to −0.25 V, with a scan rate of 0.167 mV/s. After the polarization experiment, the Epit value was determined based on when the current density rapidly increased, and the Erp value was determined based on when the current density completely decreased during the reverse scan process. After completion of the experiment, the pit shape was observed under a microscope.

## 3. Results and Discussion

### Microstructure and Component Behavior

**Figure 2:**
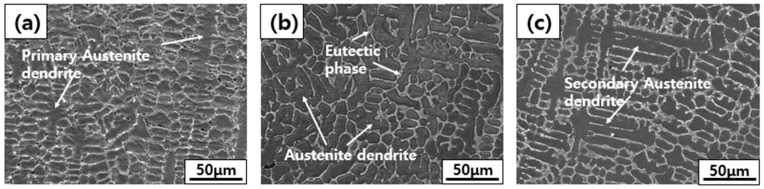
Microstructures of BSS WMs using various filler wires: (**a**) 308L, (**b**) 309L, and (**c**) 310 WMs.

Figure 2 shows the results of observing the microstructure of the BSS weld metals (WMs) using various filler wires. All weld metals were re-dissolved, and solidified areas were observed as two phases consisting of austenite dendrites and interdendritic regions formed by the eutectic reaction. A dendrite is formed when the molten WM begins to solidify owing to the high temperature during the welding process. After the initial solidification nucleus was created, it grew from the fusion line toward the center of the WM, preferentially forming primary dendrites. When this primary dendrite grows, smaller branches begin to form along the direction in which the heat escapes from the surrounding area; these branches are called secondary dendrites [18]. As the preceding branches grow preferentially, the temperature gradient decreases, and the remaining liquid metal cools uniformly in various directions in an increasingly narrow space, forming an equiaxed dendrite [19]. Secondary dendrite arm spacing (SDAS) refers to the spacing between secondary dendrites, which has a substantial effect on the mechanical properties. In general, the smaller the gap, the more it acts as a fine-grain boundary, thereby improving the strength and hardness [20,21]. The interdendritic region formed through a eutectic reaction starts to nucleate and grow simultaneously at the same time as dendrites in the liquid weld pool. The interdendritic regions of all the WMs using the three types of filler wires were formed in a very fine and continuous network-like distribution owing to the high cooling rate. The phase fraction and size of each WM were different, but the same microstructure was formed in all WMs.

**Figure 3:**
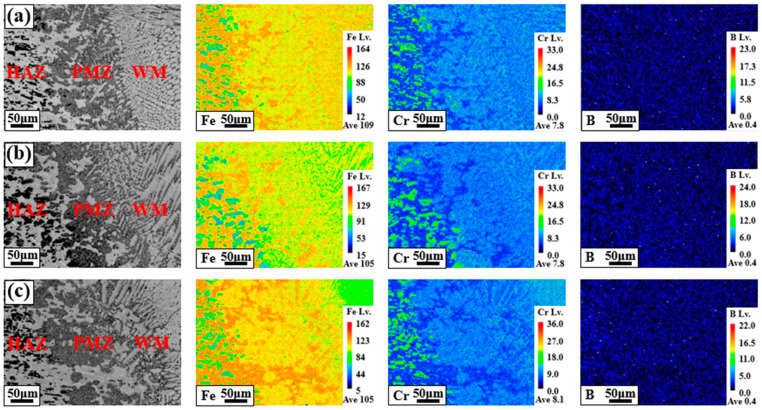
Main component segregation behaviors of BSS welds using various filler wires: fusion line areas of (**a**) 308L, (**b**) 309L, and (**c**) 310.

Figure 3 shows the main component segregation behaviors of each region [heat-affected zone (HAZ)/partially melted zone (PMZ)/WM] in the BSS welds using various filler wires. The Fe, Cr, and B components, which are the main components of each region of the BSS welds, were analyzed at a low magnification (×300). In the mapping results in Figure 3a–c, the left side corresponds to the area close to the HAZ, the center to the PMZ, and the right side to the WM. In the HAZ, a microstructure composed of a matrix and irregularly shaped particles was observed. The particles were highly enriched with Cr and B compared to the matrix, whereas the Fe component was mainly concentrated in the matrix. The color difference between the Cr and Fe components in the HAZ was more pronounced than that in the PMZ and WM, indicating significant segregation. As a result of evaluating the characteristics of BSS in a previous study, which had low corrosion resistance due to consuming the Cr component in the matrix and precipitating boride [20], it was found that the relatively dark particles in the HAZ were borides composed of Fe–Cr–B. When the WM reached the PMZ, the irregular borides in the HAZ disappeared, and these results were also confirmed in the mapping analysis results. In addition, although B components were not added to all the filler wires, as shown in Table 2, B components were observed in the PMZ and WMs of all welds. This is because the B components in the HAZ were diluted in the FZ owing to the welding heat during the welding process.

**Figure 4:**
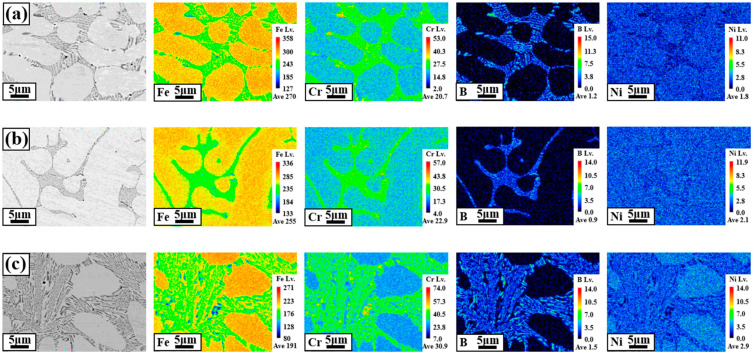
EPMA mapping results of the WMs using various filler wires: (**a**) 308L, (**b**) 309L, and (**c**) 310 WMs.

Figure 4 shows the results of the EMPA mapping to analyze the component segregation of the WM in BSS welds using various filler wires. The observation point for each WM was 1/2 t from the center of the welds. To determine the correlation between component segregation and corrosion characteristics, the Fe, Cr, B, and Ni components were analyzed at a high magnification (×3000). The mapping results in Figure 4a–c show that the microstructure consists of two phases formed by the dendritic core (DC) and interdendritic (ID) regions containing eutectic reactions. In all WMs using the three different types of filler wires, the Fe and Ni components were concentrated mainly in the DC. However, in the case of Ni mapping, it was relatively less concentrated in the DC than in Fe, and a somewhat uniform composition was formed. The segregation of the Cr and B components was concentrated in the ID region containing the eutectic reaction. Additionally, the ID region containing the eutectic reaction is composed of Fe–Cr–B components, similar to the boride in the HAZ, as shown in Figure 3.

Table 3 shows the results of the chemical composition analysis of the WMs obtained through ICP analysis of the BSS welds using various filler wires. The Cr content of the filler wire gradually increased from 308L to 310L, showing the same trend as the WMs. In addition, although component B was not added to the filler wire, it was confirmed that component B was contained in the WM, as it was diluted by the welding heat during the welding process in the HAZ. However, because the dilution amount of the B component in the HAZ was different, the content of B was the highest in the 308L WM and lowest in the 309L WM.

**Figure 5:**
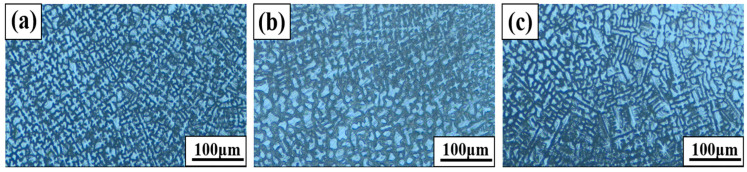
Sensitization results of the WMs using various filler wires: (**a**) 308L, (**b**) 309L, and (**c**) 310 WMs.

Figure 5 shows the results of the sensitization evaluation of BSS WMs using various filler wires. The evaluation method is broadly divided into three structural stages: step, dual, and itch. The step structure is a state in which intergranular attack does not occur at the grain boundaries. A dual structure is a state in which an intergranular attack occurs at some grain boundaries, but no single grain is completely surrounded. A ditch structure is a state in which one or more grains are completely surrounded by intergranular attack. ID ditches occurred in all the WMs using the three different types of filler wires. This indicates that intergranular corrosion occurred in the ID regions and that these regions were susceptible to corrosion. These results indicate that ID ditches occurred in all the WMs because the passive film became thin owing to the segregation of the Cr component in the ID region containing the eutectic reaction shown in the previous mapping results. As a result, ID ditches were formed in all the WMs, and the more ID regions there were, the more the Cr-depleted areas increased, leading to a higher corrosion susceptibility. Therefore, the corrosion susceptibility is related to the dendrite growth behavior.

**Figure 6:**
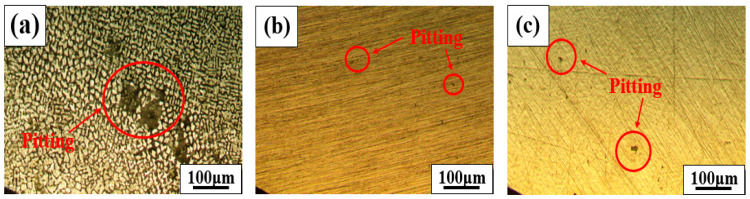
Pitting corrosion resistance results of the WMs using various filler wires: (**a**) 308L, (**b**) 309L, and (**c**) 310 WMs.

Figure 6 shows the pitting corrosion sensitivity measurement results for the BSS WMs using various filler wires. As shown in Figure 6a, general corrosion occurred on the 308L WM surface, revealing an overall dendritic structure. In addition, multiple large pits are observed, confirming the occurrence of pitting corrosion. However, as shown in Figure 6b,c, general corrosion did not occur, and the pit morphology was relatively small. The pitting corrosion resistance of stainless steel is generally assessed using the pitting resistance equivalent number (PREN), as shown in Equation (1):

PREN = % Cr + 3.3%Mo + 16N(1)

Because BSS WMs using the three types of filler wires do not contain Mo, their PREN values depend solely on the Cr content. The WMs had PREN values of 20.25 for 308L, 23.27 for 309L, and 24.85 for 310, indicating superior pitting corrosion resistance depending on the Cr content. In the case of the 308L WM, owing to its lower Cr content compared with other WMs, a relatively thin passive film formed, resulting in both general corrosion and a very large pit morphology [22]. However, the 310 WM had a higher Cr content than the 309L WM, resulting in a higher PREN value, but it exhibited larger and more numerous pits, leading to lower pitting corrosion resistance compared to the 309L WM. This is because the 310 WM, which has a high B content, consumed more Cr than the 309L WM in the previous ICP results because of the mechanism of consuming Cr and B in the BSS weld zone to form a eutectic structure. Therefore, the pitting corrosion resistance in the BSS welds did not follow typical PREN values owing to the segregation of Cr.

**Figure 7:**
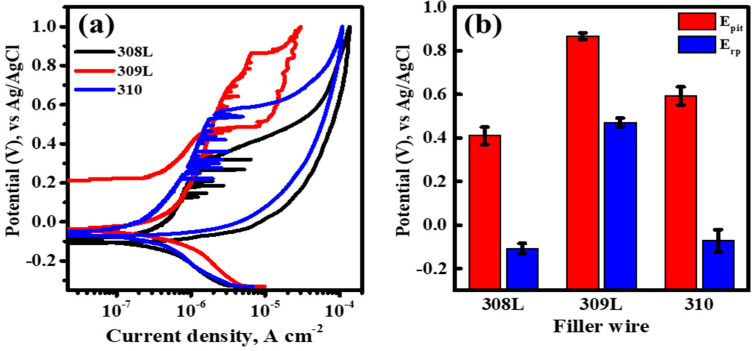
(**a**) Cyclic polarization curves of BSS WMs using various filler wires measured in 50 ppm NaCl solution, at 27 °C, and with potential sweep rate of 0.167 mVs^−1^. (**b**) Pitting and repassivation potential (E_pit_ and E_rp_, respectively) of WM along the filler wire measured through the cyclic polarization curve.

Figure 7a shows the polarization behavior and pitting corrosion resistance of the BSS WMs with various filler wires in a 50 ppm NaCl solution as a cyclic polarization curve obtained from the potentiodynamic polarization experiments. The corrosion potential (E_corr_) values of all WMs in the 50 ppm solution were similarly formed in the range of −0.1 to 0 V vs. Ag/AgCl. Additionally, a passive region was formed between the corrosion potential and pitting potential (E_pit_). However, in the passive region, metastable peaks appeared before the pitting potential was reached. These metastable peaks indicate unstable passive formation owing to the growth of pitting nuclei at lower potentials. Although all WMs with the three types of filler wires showed similar polarization behavior up to the passive region, there were significant differences in the Epit values and repassivation potential (Erp), which is the potential at which the metal returns to a passive state after pitting corrosion. Generally, higher E_pit_ values indicate better resistance to pitting corrosion, allowing the metal to maintain its passivity even under extremely corrosive conditions. In addition, higher E_rp_ values indicate that the metal can repassivate at higher potentials after pitting occurs. Figure 7b shows the E_pit_ and E_rp_ values measured using the cyclic polarization curves. Regardless of the PREN value, both the E_pit_ and E_rp_ values were the highest for the 309L WM, indicating the highest resistance to pitting corrosion and the fastest repassivation after pitting occurred. The next highest values were observed for 310 WM, which had a higher Cr content, and the lowest values were found for 308L WM. Consistent with the results of the pitting tests shown in Figure 6, the pitting resistance of the BSS WMs in the 50 ppm NaCl solution did not follow the PREN values. These results align with the findings of Ha’s study [14], which reported that Cr segregation caused by boride formation diminishes the pitting resistance of stainless steel. Similarly, this study confirmed that Cr and B predominantly segregate within the ID regions containing eutectic reactions, forming Cr-depleted zones that serve as primary sites for pitting corrosion. This demonstrates that the pitting resistance of BSS weld metals is not solely governed by the PREN value but is significantly affected by the segregation of Cr and B during the welding process.

**Figure 8:**
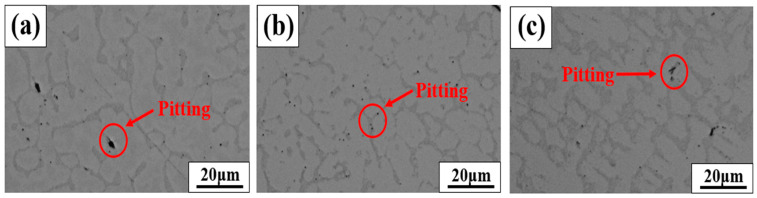
Results of localization of pits and microstructural observations after cyclic polarization testing of BSS WMs with various filler wires: (**a**) 308L, (**b**) 309L, and (**c**) 310 WMs.

Figure 8 shows the pits and microstructures after the cyclic polarization experiments on the BSS WM using various filler wires. In all BSS WMs that used the three types of filler wires, most of the pits, both large and small, occurred between the eutectic structure and dendrite interface. However, a few pits are observed in the ID regions. The pits observed in the ID regions are indicated by the red circles. The ID regions containing eutectic reactions were composed of Fe–Cr–B, resulting in a concentrated distribution of Cr in these regions, as shown in Figure 4. However, Cr-depleted areas occur at the interfaces of the ID regions containing eutectic reactions, causing the pits to concentrate at these interfaces. Consequently, to form a eutectic structure, Cr was depleted around it, making these areas more susceptible to pitting corrosion. Therefore, it can be seen that the corrosion resistance of each WM is related to the Cr/B content and the fraction of ID regions containing eutectic reactions.

**Figure 9:**
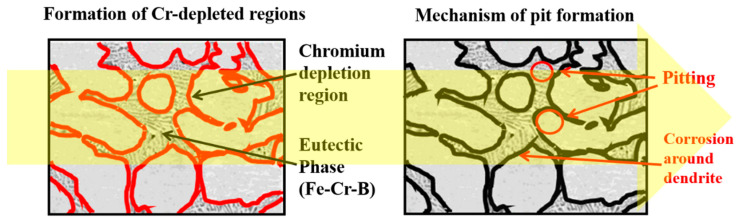
Schematic diagram of pit formation after cyclic polarization test.

Figure 9 shows a schematic diagram showing the pit formation location formed after cyclic polarization testing. In the ID region, which includes the eutectic reaction, the Cr component was concentrated and segregated. However, at the interface between the ID region and the dendrite, a Cr-depleted region was formed, leading to a specific area where the corrosion characteristics deteriorated. Therefore, the results shown in Figure 8 confirmed that the pits formed during the cyclic polarization experiment occurred either in the ID region, including the interface between dendrites, or at the location of the dendrite adjacent to the Cr-depleted region. The location where pits are formed is clearly shown in the schematic diagram in Figure 9, and the corrosion mechanism of BSS welds can be elucidated.

To apply BSS as a spent nuclear fuel storage container material, its neutron absorption capacity is important; however, its weldability and corrosion resistance are also important factors. Based on the results of this study, the use of filler metals with low boron content is expected to be effective in improving the weldability and corrosion resistance of BSS materials. However, the boron content of the filler metal must be carefully considered because a lower boron content reduces the neutron absorption capacity. Therefore, for BSS to be used as a spent nuclear fuel storage container material, the boron content of the filler metal must be carefully considered. In this study, the weldability and corrosion properties of BSS were evaluated for use as a nuclear material. As the boron content increased, the fraction of the Cr-depleted region within the WM increased, which negatively affected the corrosion characteristics of the BSS weld. These research results can serve as a basis for developing filler wires and corrosion evaluation technologies for BSS welding.

## 4. Conclusions

In this study, GTA welding was conducted using BSS base metal with a 1.62 wt.% B content and various filler wires. This study focused on evaluating the corrosion resistance of WMs in relation to their microstructural and compositional behaviors.

(1) Eutectic reactions involving the B component were observed in the ID regions of all WMs, resulting from the dilution of the B component of the BM into the WM during welding.
(2) As a result of the pitting corrosion sensitivity, 308L WM with a high fraction of B components (0.64 wt.%) showed the highest corrosion sensitivity.
(3) As a result of the polarization test, 309L WM with a low fraction of B components (0.20 wt.%) exhibited the highest corrosion resistance (E_pit_; 0.87 V and E_rp_; 0.45 V), indicating the fastest repassivation after pitting formation.
(4) Component segregation of boron in the WM has a negative impact on the corrosion resistance of welded specimens because it causes Cr-depleted areas.
(5) Using a filler metal with a low boron content is expected to effectively improve the weldability and corrosion resistance of BSS materials.
(6) When using BSS as a nuclear material, it is essential to carefully evaluate and control the boron content to ensure optimal weldability and corrosion resistance.
(7) These research results can serve as a basis for developing filler wires and corrosion evaluation technologies for BSS welding.

## Figures and Tables

**Table 1:** Chemical composition of the base metal and filler wires used in this study.

Composition (wt.%)		C	Mn	Si	Cr	Ni	B	N	Co
Base metal	304B6	0.08	2.00	0.75	19.00	13.50	1.62	0.10	-
Filler wire	308L	0.022	1.84	0.39	19.75	9.66	-	0.027	0.164
	309L	0.011	1.54	0.47	23.12	13.78	-	0.055	0.184
	310	0.091	1.56	0.48	26.63	21.06	-	0.019	0.236

**Table 2:** Welding conditions used in the present study.

Pass	Current (A)	Voltage (V)
1	50 (base current)—20 (pulse current)	11
2~3	70	11

**Table 3:** Chemical compositions of the WMs using three types of filler wire (wt.%).

WM	C	Mn	Si	Cr	Ni	B	N	Co
308L	0.016	1.67	0.41	19.51	11.26	0.649	0.046	0.044
309L	0.010	1.99	0.42	22.53	13.51	0.202	0.046	0.120
310	0.069	1.45	0.47	24.47	19.04	0.482	0.024	0.177

## Data Availability

The original contributions presented in the study are included in the article, further inquiries can be directed to the corresponding author.
